# Novel Opportunity to Reverse Antibiotic Resistance: To Explore Traditional Chinese Medicine With Potential Activity Against Antibiotics-Resistance Bacteria

**DOI:** 10.3389/fmicb.2020.610070

**Published:** 2020-12-22

**Authors:** Ting Su, Ye Qiu, Xuesi Hua, Bi Ye, Haoming Luo, Da Liu, Peng Qu, Zhidong Qiu

**Affiliations:** ^1^College of Pharmacy Changchun University of Chinese Medicine, Changchun, China; ^2^College of Literature, Science and Arts University of Michigan, Ann Arbor, MI, United States; ^3^National Cancer Institute, Frederick, MD, United States

**Keywords:** antibiotic-resistant bacteria, traditional Chinese medicine, active ingredients, reversion mechanism, multi-drug resistance

## Abstract

Antibiotic resistance is becoming significantly prominent and urgent in clinical practice with the increasing and wide application of antibacterial drugs. However, developing and synthesizing new antimicrobial drugs is costly and time-consuming. Recently, researchers shifted their sights to traditional Chinese medicine (TCM). Here, we summarized the inhibitory mechanism of TCM herbs and their active ingredients on bacteria, discussed the regulatory mechanism of TCM on antibiotic-resistant bacteria, and revealed preclinical results of TCM herbs and their active components against antibiotic-resistant bacteria in mouse models. Those data suggest that TCM herbs and their effective constituents exhibit potential blockage ability on antibiotic-resistant bacteria, providing novel therapeutic ideas for reversing antibiotic resistance.

## Introduction

Bacterial infection is an acute systemic infection caused by pathogenic bacteria or pathogens that proliferate through invading the blood circulation system. The discovery of antibiotics has been one of the most important medical interventions in the history of global health. Antibiotics have been applied to reduce the morbidity and mortality caused by bacterial infections. Antibiotics therapies on infection are shown to be progressive for a period of time followed by the loss or decreasing effect on bacterial prevention due to the development of antibiotic resistance, which refers to reduced or even disappeared efficacy of drugs against bacterial infection after those drugs are repeatedly used. Antibiotic resistance to bacterial pathogens leads to prolonged hospital stays, higher medical costs, complex complications, and increasing mortality rate in patients. Advanced medical therapeutic methods, such as organ transplantations, chemotherapy and surgeries, have become much more risky without effective antibiotics against bacterial infections (Carver et al., 2017; Hollyer and Ison, 2018). Therefore, it is dramatically important for human health to effectively prevent the spread of antibiotic-resistant bacteria.

A number of researchers have studied the mechanism of antibiotic resistance to guide clinical drug use, further avoiding the treatment delay or failure caused by the use of non-sensitive drugs and averting the emergence of new antibiotic-resistant bacteria. In the meanwhile, the investigators try to explore novel effective bacterial inhibitors. The contradiction between the slow development of new antibiotics and the rapid increase of antibiotic-resistant strains is non-negligible. Some scientists have turned their attention to traditional Chinese medicine (TCM). TCM herbs and their active ingredients have been found to exert multiple antibacterial roles and effective therapeutic effects on antibiotic-resistant bacteria. Therefore, the rescue roles of TCM on antibiotic resistance have become an international research hotspot in recent years. In the review, we summarize the inhibitory functions and reversion mechanisms of TCM herbs and their active ingredients against antibiotic resistance, providing an alternative path for finding new treatments against bacterial infections.

## Mechanisms of Antibiotic Resistance

**FIGURE 1:**
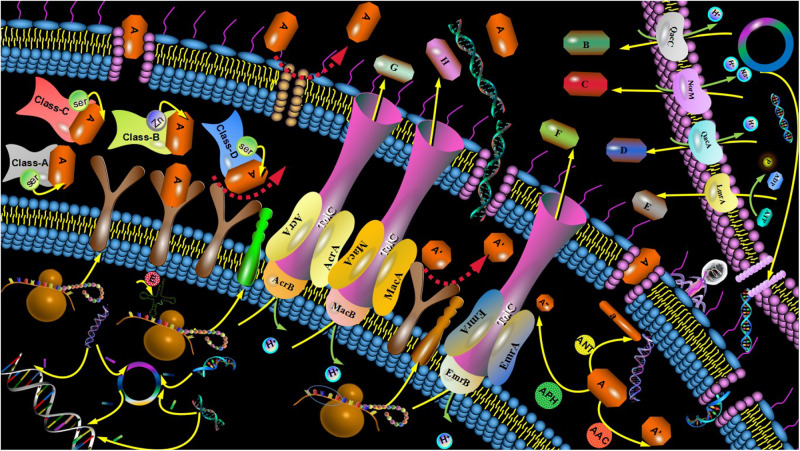
Mechanism of antibiotic resistance. The mechanisms of antibiotic resistance included both genetic and mechanistic basis of antibiotic resistance. The drug-resistant mutant gene was performed between G+ and G- bacteria and transferred to the next generation strain. Sensitive strains acquired resistant genes from plasmids of resistant strains, bacteriophages, or free DNA. Porin alterations induced by the type shift of expressed porins, the change in the expression level of porin and impairment of the porin function reduce the permeability of the bacterial outer membrane. Drug did not recognize drug targets due to structure changes of drug targets or enzymatic alterations of the binding site, leading to drug resistance. The BLs were divided into four categories: A, B, C, and D. The A, C, and D enzymes known as SBLs. Class B enzymes known as MBLs. Common AMEs include AAC, AAD, ANT, and APH. Efflux system can be divided into 5 superfamilies: MFS, RND, ABC, MATE, and SMR according to the different structures of the active exportation systems. Efflux system was mainly composed of three parts: membrane fusion protein, efflux transporter protein and outer membrane protein.

It is difficult for us to solve the problem of multidrug-resistance unless we understand the mechanisms by which the resistance is initiated (Frieri et al., 2017). The main mechanism of antibiotic resistance not only includes both genetic and mechanistic basis of antibiotic resistance, but also involves in bacterial biofilm (BBF) formation (Roy et al., 2018). The genetic basis of antibiotic resistance is involved in both intrinsic and acquired resistance (Olaitan et al., 2014), while the mechanistic basis of antibiotic resistance includes the modifications of the antibiotic molecule, changes in target sites, decreased antibiotic penetration, and efflux pumps (Munita and Arias, 2016; Figure 1).

Intrinsic resistance, one of genetic basis of antibiotic resistance, refers to the innate insensitivity of bacteria to antimicrobial agents, which is determined by the biological characteristics of bacteria, such as structure and metabolic mechanism. Gram-negative (G-) bacteria, such as *Escherichia coli*, are generally not sensitive to neomycin antibiotics, *Streptococcus mutans* (*S. mutans*). *Staphylococcus aureus* (*S. aureus*), while Gram-positive (G+) bacteria are less sensitive to streptomycin. The inherent drug resistance is mainly caused by the drug-resistant gene located on the chromosome of the bacteria, which passes from generation to generation with specificity.

Acquired resistance occurs when an antibiotic-sensitive organism becomes resistant through gene acquisition or mutations (Arzanlou et al., 2017). Sensitive strains may acquire resistant genes from plasmids of resistant strains, bacteriophages or free DNA. Drug-resistant genes are transferred through transformation, transduction, and conjugation (Munita and Arias, 2016).

Bacteria can produce enzymes that modify antibiotic molecules either by destroying the molecules or inactivating the drug with specific chemicals to prevent antibiotics from interacting with the target. Antibiotic-resistant bacteria also produce modification enzymes and hydrolases to prevent various antimicrobial agents from entering the cells. Modification enzymes, known as synthetases, are mainly used to reduce the activity of antibiotic groups and decrease the binding rate with corresponding targets. For instance, the bacteria produce the enzymes that inactivate aminoglycoside antibiotics. Those aminoglycoside modification enzymes include acetylase (AAC), adenylase (AAD), nucleotidase (ANT), and phosphorylase (APH; Ramirez and Tolmasky, 2017). Hydrolases can directly destroy the active components of antibiotics. β-lactamase (BLs) as one kind of hydrolases, can hydrolyze the active molecules of lactamides to make bacteria resistant to lactamides. According to their amino acid sequence and mechanisms of action, BLs are divided into four categories: A, B, C, and D. The A, C, and D enzymes are known as serine β-lactamase (SBLs), whose catalytic hydrolysis β-lactam ring active centers rely on Serine (Ser). Class B enzymes, known as metal-β-lactamase (MBLs), are dependent on the presence of zinc ions (Figure 1). In addition, antibiotic-targeted bacteria is not identified by the active groups of antibiotics due to the structural modification of those bacteria, such as the point mutations in genes that encoded the target site and enzymatic alterations of the binding site (Munita and Arias, 2016).

The plenty numbers of antibiotics, such as aminoglycoside molecules, within the cell damaged the cell wall and cell membrane to promote a high rate of uptake that ultimately resulted in cell death (Garneau-Tsodikova and Labby, 2016). Bacteria resist antibiotics by reducing the permeability of extracellular membrane of bacteria cells or using efflux pumps to remove antibiotics from the entrance of the cell (May and Grabowicz, 2018). Those non-specific alterations in permeability give rise to cross-resistance to different types of antibiotics.

The efflux pump systems reduced the intracellular concentrations of antimicrobial agents to make bacteria more recalcitrant to treatment and was divided into five super families based on their structures (Bruns et al., 2017). Those five super families included major facilitator superfamily (MFS), resistance nodulation division superfamily (RND), ATP-binding cassette family (ABC), multidrug and toxic compound extrusion family (MATE), and small multidrug resistance (SMR; Fernandez and Hancock, 2013; Mahmood et al., 2016; Figure 1).

Biofilm was described as the complex sessile communities of microbes that adhered to the surface or stretched firmly in the extracellular matrix, wrapping around the bacteria to make them resistant to antibacterial treatments (Roy et al., 2018). The main resistance mechanisms of BBF include penetration restrictions (Singh et al., 2017), nutrition restrictions, and signal transmission between bacteria, such as Quorum Sensing (QS; Mukherjee and Bassler, 2019).

## TCM Suppresses Antibiotic-Resistant Bacteria

There is a resourceful of medicinal plants in the global scale, in which 400 species of TCM herbs are included. The application of those TCM herbs on the prevention and treatment of diseases, including infection and cancer, has been practiced for several thousand years. There are increasing evidences that TCM herbs, including monomers of TCM (Table 1) and extracts of TCM (Table 2), exhibit obvious antibacterial ability and enhance the activity of antibiotics (Ma et al., 2010), and some of them diminish antibiotic resistance (Wu et al., 2008). TCM treatment on infection diseases have many advantages, such as abundant resources, moderate price, multi-component, multi-target, and medical synergism. Therefore, TCM treatment may be one of the effective methods to solve the problem of antibiotic resistance.

**TABLE 1:** Active ingredients of TCM herbs.

**Scientific name**	**Compound**	**Active against**	**Combination antibiotic**	**References**
*Alpinia galanga* (L.) Swartz	1′-Acetoxychavicol, Galangin	*R-* -*E. faecalis, R-S. typhi, R-P. aeruginosa, R-E. coli*, -*R-Bacillus cereus, R-S. aureus*		Lathaa et al., 2009
*Arnebia euchroma* (Royle) Johnst.	Isovalerylshikonin	*R-S. aureus*	Streptomycin	He J. M. et al., 2018
*Coptis chinensis* Franch	Berberine, 5′-Methoxyhydnocarpin, Berberine, Coptisine, Palmatine, Epiberberine, Jatrorrhizine	*MRSA, S. aureus*		Yu et al., 2005; Luo et al., 2014
*Coriandrum sativum* L.	Linalool	*A. baumannii*		Alves et al., 2016
*Daphne genkwa* Sieb.et Zucc.	Tiliroside, Pinoresinol, Magnatriol B, Momorcharaside B	*MRSA*		Kuok et al., 2017
*Glycyrrhiza glabra* L.	Glycyrrhizic acid	*R-E. faecium*	Gentamicin	Schmidt et al., 2016
*Hypericum densiflorum, Hypericum ellipticum, Hypericum prolificum, Hypericum punctatum*	2-geranyloxy-1-(2-methylpropanoyl) phloroglucinol, 3-geranyloxy-1-(2-methylbutanoyl) phloroglucinol, 2-geranyloxy-4,6-dihydroxybenzophenone, 3-prolificin A	*MRSA*		Sarkisian et al., 2012
*Illicium verum* Hook.f.	(E)-Anethole, Anisyl acetone, Anisyl alcohol, Anisyl aldehyde	*MRSA, A. baumannii, P. aeruginosa*		Jyh-Ferng et al., 2010
*Magnolia officinalis*	Tiliroside, Pinoresinol, Magnatriol B, Momorcharaside B	*MRSA*	Oxacillin	Kuok et al., 2017
*Phellodendron amurense* Rupr.	Berberine	*MRSA, P. aeruginosa*	Imipenem	Su and Wang, 2017
*Piper nigrum* L.	Piperine	*R-M. tuberculosis, S. aureus*	Mupirocin	Mirza et al., 2011
*Rosa canina* L. (rose red)	Corilagin, Tellimagrandin I	*MRSA*	Benzyl Penicillin	Shiota et al., 2004
*Silybum marianum (L.) Gaertn.*	Silybin	*MRSA*	Ciprofloxacin	Wang et al., 2018
*Toddalia asiatica* (Linn) Lam	Chelerythrine	*MRSA, R-S. aureus*		He N. et al., 2018
*Verbena officinalis* L.	Tiliroside, Pinoresinol, Magnatriol B, Momorcharaside B	*MRSA*	Oxacillin	Kuok et al., 2017

**TABLE 2:** Antibacterial activity of TCM extracts.

**Scientific name**	**Extracts**	**Active against**	**References**
*Allium sativum* L.		*E. coli*	Bruns et al., 2017
*Areca catechu* L.		*C. violaceum; P. aeruginosa*	Koh and Tham, 2011
*Armeniaca vulgaris* Lam.		*C. violaceum; P. aeruginosa*	Koh and Tham, 2011
*Berberis aristata*		*R-E. coli*	Thakur et al., 2016
*Cuminum cyminum* L.		*MRSA*	Kakarla et al., 2017
*Dracontomelon dao* (Blanco) Merr. Rolfe	Flavonoids and phenolic	*R-E. coli*	Xu et al., 2019
*Herba patriniae*	Extract	*P. aeruginosa*	Fu et al., 2017
*Holarrhena antidysenterica*		*R-E. coli*	Thakur et al., 2016
*Hypericum perforatum* L.	Ethanol or water extract	*E. faecalis*	Süntar et al., 2015
*Imperata cylindrica* (L.) Beauv.		*C. violaceum; P. aeruginosa*	Koh and Tham, 2011
*Lagerstroemia speciosa* (L.) Pers.	Extract	*P. aeruginosa*	Singh et al., 2012
*Momordica charantia* L.	Ethanol extract	*MRSA*	Coutinho et al., 2010
*Nelumbo nucifera*		*C. violaceum; P. aeruginosa*	Koh and Tham, 2011
*Panax notoginseng* (Burkill) F. H. Chen ex C. H.		*C. violaceum; P. aeruginosa*	Koh and Tham, 2011
*Pilgerodendron uviferum*	Essential oils, petroleum ether extracts, dichloromethane extract	*S. aureus*	Espinoza et al., 2019
*Plumbago zeylanica* L.	Flavonoids, saponins, naphthoquinone	*R-S. paratyphi, R-S. aureus, R-E. coli, R-S. dysenteriae*	Beg and Ahmad, 2000
*Prunella vulgaris* L.		*C. violaceum; P. aeruginosa*	Koh and Tham, 2011
*Psoralea corylifolia*	Ethanol extract	*MRSA; L. monocytogenes*	Li et al., 2019
*Punica granatum* L.		*C. violaceum; P. aeruginosa*	Koh and Tham, 2011
*Rhizophora mucronata*	Methanol extract	*P. aeruginosa*	Annapoorani et al., 2013
*Rhizophora apiculata*	Methanol extract	*P. aeruginosa*	Annapoorani et al., 2013
*Sanguisorba officinalis* L	Ethanol extract	*MRSA*	Chen et al., 2015
*Withania somnifera* L.	Methanol extract, ethanol extract, butanol extract	*MRSA*	Datta et al., 2011
*Zingiber officinale* Rosc.	Extract	*P. aeruginosa*	Kim and Park, 2013

### Inhibition Activity of Monomer of TCM Herbs on Antibiotic-Resistant Bacteria Activity

The single components of some TCM herbs exert antibacterial activity. Alves et al. demonstrated the antimicrobial activity of the major oil compound (linalool) of *Coriandrum sativum* against *Acinetobacter baumannii* (*A. baumannii*), and evaluated its roles on planktonic cells and biofilms of *A. baumannii* on different surfaces, as well as its effect on adhesion and QS. Linalool inhibited biofilm formation, dispersed established biofilms of *A. baumannii*, changed the adhesion of *A. baumannii* to surfaces and interfered with the QS system (Alves et al., 2016). In addition, the multiple elements of the same TCM herbs display similar antibacterial activity. (E)-anethole, anisyl acetone, anisyl alcohol and anisyl aldehyde, identified from the extracts of *Illicium verum*, exhibit the synergistic antibacterial activity against 67 clinical antibiotic-resistant isolates, including 27 *A. baumannii*, 20 *Pseudomonas aeruginosa* (*P. aeruginosa*), and 20 MAS, indicating that those three compounds might be the active ingredients of *Illicium verum* with antibacterial activity (Jyh-Ferng et al., 2010; Table 1).

For different TCM herbs in the same family, there may be identical antibacterial active ingredients. Sarkisian et al. isolated five secondary metabolites from the same family species *Hypericum densiflorum, Hypericum ellipticum, Hypericum prolificum*, and *Hypericum punctatum*, which all inhibited bacterial growth and biofilm production. The five compounds, including 3-geranyl-1-(2-methylpropanoyl) phloroglucinol, 3-geranyl-1-(2-methylbutanoyl) phloroglucinol, 2-geranyloxy-1-(2-methylpropanoyl) phloroglucinol, 2-geranyloxy-1-(2-methylbutanoyl) phloroglucinol, and 2-geranyloxy-4,6-dihydroxybenzophenone, displayed the inhibitory activity against the G- bacteria and biofilm formation at low concentrations (Sarkisian et al., 2012; Table 1).

Some Chinese herbal ingredients may be used as sensitizers to improve the sensitivity of antibiotic-resistant bacteria to medical antibiotics. Piperine isolated from black pepper was shown to enhance antimicrobial activity of mupirocin against *S. aureus* strains including MASA through the inhibition of efflux of ethidium bromide (Mirza et al., 2011). In addition, Schmidt et al. (2016) revealed therapeutic potential of glycyrrhizic acid in co-application with gentamicin for defined local bacterial infections caused by vancomycin-resistant *Enterococcus* strains, indicating that glycyrrhizic acid improve the antimicrobial activity of gentamicin to antibiotic-resistant bacteria (Table 1).

### Inhibition Roles of Extracts of TCM Herbs on Drug-Resistant Bacteria Activity

Researchers isolate active extracts of antibiotic-resistant bacteria from TCM herbs through applying different solvents, such as essential oil, water extract, and ethanol extract. Coutinho et al. (2010) demonstrated that the ethanol extract of *Momordica charantia L.* (Cucurbitaceae) displayed the antibiotic activity against Methicillin-resistant *Staphylococcus aureus* (*MRSA*) strain, indicating the potentiating effect of the ethanol extract on aminoglycosides. Even though there are different extracts from same TCM hebs, they exert similar activity. The methanol extract, ethanol extracts and butanol extract fractions from *Withania somnifera* (L) Dunal was found to be effective against the multi-drug resistant (MDR) *S. aureus* strains (Datta et al., 2011). However, for some of TCM herbs, different extracts of same TCM herbs may exhibit the distinct antimicrobial activity for different antibiotic-resistant bacteria. The ethanol extract of *Hypericum perforatum L.* exert strong antimicrobial activity against *S. mutans, S. sobrinus, Lactobacillus plantarum (L. plantarum)*, and *Enterococcus faecalis* (*E. faecalis*). Its water extracts display strong antibacterial activity against *S. sobrinus* and *L. plantarum* and exerted moderate activity against *S. mutans* and *E. faecalis*. Both ethyl acetate and n-butanol extracts from *Hypericum perforatum L.* exhibit antimicrobial activity against *L. plantaru* (Süntar et al., 2015; Table 2).

The chemical components of TCM extracts are complicated. Even though some TCM herbs can prevent antibiotic-resistant bacteria, their bacteriostatic concentrations are relatively high, resulting in the decrease of clinical practicability. Therefore, many researchers identified the active components of TCM extracts to promote the application of TCM on antibiotic-resistant bacteria using advanced techniques. Luo et al. (2014) utilize chemical fingerprinting to find that the functional components of *Rhizoma coptidis* (*Coptis chinensis Franch.* Huanglian in Chinese) are alkaloids, which display the ability against the drug resistance induced by *NorA* gene. Ethyl acetate extracts deriving from the leaves of *Dracontomelon Dao* (Blanco) *Merr. & Rolfe* exhibit obvious antibacterial activity on ampicillin-resistant *E. coli*. Furthermore, Xu et al. (2019) revealed that flavonoids and phenolic acids isolated from the ethyl acetate extracts were active ingredients using liquid chromatography–mass spectrometry (Table 2).

## Inhibitory Mechanism of TCM on Antibiotic Resistance

Traditional Chinese medicine herbs and their active components have great potentials and advantages in rescuing drug-resistant bacteria (Table 3). The mechanism is mainly manifested in the following aspects. TCM herbs block the genetic basis of bacterial resistance by eliminating resistant plasmids, and the mechanical basis of bacterial resistance by increasing the permeability, inhibiting the efflux pump, modifying the antibiotic molecule and changing drug targets. Those TCM herbs and their compounds also prevent antibiotic resistance by destroying the BBF.

**TABLE 3:** Inhibitory Mechanism of TCM Herbs on antibiotic resistance.

**Mechanism of action**	**Scientific name**	**Active against**	**References**
To induce R-plasmid	*Alpinia galanga* (L.) Swartz	*R-S. typhi, R-P. aeruginosa, R-E. coli, R-Bacillus cereus, R-S. aureus*	Lathaa et al., 2009
	*Plumbago zeylanica* L.	*R-S. paratyphi, R-S. aureus, R-E. coli, R-S. dysenteriae*	Beg and Ahmad, 2000
To increase in membrane permeability	*Berberis aristata*	*R-E. coli*	Thakur et al., 2016
	*Camellia sinensis*	*R-E. coli*	Thakur et al., 2016
	*Rhodomyrtus tomentosa*	*MRSA, R-E. faecium*	Zhao et al., 2019
	*Toddalia asiatica* (Linn) Lam	*MRSA, R-S. aureus*	He N. et al., 2018
To inhibit the efflux *EmrD-3* pump *LmrS NorrA NorrA/abcA EtBr MexXY-OprM*	*Allium sativum* L.	*K. pneumoniae, A. baumannii, P. aeruginosa, S. aureus, S. epidermidis, V. cholerae*	Bruns et al., 2017
	*Arnebia euchroma* (Royle) Johnst.	*R-S. aureus*	He J. M. et al., 2018
	*Cuminum cyminum* L.	*MRSA*	Kakarla et al., 2017
	*Coptis chinensis Franch.*	*MRSA*	Luo et al., 2014
	*Psoralea corylifolia*	*MRSA;Listeria monocytogenes*	Li et al., 2019
	*Pilgerodendron uviferum*	*S. aureus*	Espinoza et al., 2019
	*Phellodendron amurense* Rupr.	*P. aeruginosa*	Su and Wang, 2017
	*Silybum marianum (L.) Gaertn.*	*MRSA*	Wang et al., 2018
To suppress the PBPs	*Daphne genkwa* Sieb.et Zucc.	*MRSA*	Kuok et al., 2017
	*Magnolia officinalis*	*MRSA*	Kuok et al., 2017
	*Rosa canina* L. (rose red)	*MRSA*	Shiota et al., 2004
	*Verbena officinalis* L.	*MRSA*	Kuok et al., 2017
To block β-lactamase	*Camellia sinensis*	*MRSA*	Stapleton et al., 2004
	*Coptis chinensis Franch. (Ranunculaceae)*	*MRSA*	Yu et al., 2005
	*Daphne genkwa* Sieb.et Zucc.	*MRSA*	Kuok et al., 2017
	*Magnolia officinalis*	*MRSA*	Kuok et al., 2017
	*Rosa canina* L. (rose red)	*MRSA*	Shiota et al., 2004
	*Verbena officinalis* L.	*MRSA*	Kuok et al., 2017
To inhibit biofilm formation *QS*	*Areca catechu* L.	*C. violaceum; P. aeruginosa*	Koh and Tham, 2011
	*Armeniaca vulgaris* Lam.	*C. violaceum; P. aeruginosa*	Koh and Tham, 2011
	*Imperata cylindrica* (L.) Beauv.	*C. violaceum; P. aeruginosa*	Koh and Tham, 2011
	*Nelumbo nucifera*	*C. violaceum; P. aeruginosa*	Koh and Tham, 2011
	*Panax notoginseng* (Burkill) F. H. Chen ex C. H.	*C. violaceum; P. aeruginosa*	Koh and Tham, 2011
	*Prunella vulgaris* L.	*C. violaceum; P. aeruginosa*	Koh and Tham, 2011
	*Punica granatum* L.	*C. violaceum; P. aeruginosa*	Koh and Tham, 2011
	*Berberis aristata*	*R-E. coli*	Thakur et al., 2016
	*Camellia sinensis*	*R-E. coli*	Thakur et al., 2016
	*Coriandrum sativum* L.	*A. baumannii*	Alves et al., 2016
	*Herba patriniae*	*P. aeruginosa*	Fu et al., 2017
	*Hypericum densiflorum*	*MRSA*	Sarkisian et al., 2012
	*Hypericum ellipticum*	*MRSA*	Sarkisian et al., 2012
	*Hypericum prolificum*	*MRSA*	Sarkisian et al., 2012
	*Hypericum punctatum*	*MRSA*	Sarkisian et al., 2012
	*Hypericum perforatum* L.	*E. faecalis*	Süntar et al., 2015
	*Lagerstroemia speciosa* (L.) Pers.	*P. aeruginosa*	Singh et al., 2012
	*Phellodendron amurense* Rupr.	*MRSA*	Yu et al., 2005
	*Rhizophora apiculata*	*P. aeruginosa*	Annapoorani et al., 2013
	*Rhizophora mucronata*	*P. aeruginosa*	Annapoorani et al., 2013
	*Sanguisorba officinalis* L	*MRSA*	Chen et al., 2015
	*Scutellaria barbata* D. Don	*R-A. baumannii*	Tsai et al., 2018
	*Zingiber officinale* Rosc.	*P. aeruginosa*	Kim and Park, 2013

### Elimination of Resistant Plasmids

The formation, transfer and transmission of resistant plasmids are important mechanisms that cause extensive antibiotic resistance, which play a major role in the dissemination of resistance genes (Laurent et al., 2018; Lerminiaux and Cameron, 2019). One of the effective mechanisms of TCM on decreasing antibiotic resistance may be to inhibit the transfer of resistant plasmids or eliminate those plasmids. 1′-Acetoxychavicol acetate from ***Alpinia galanga (L.) Swartz*** disposes the ability to decrease plasmid-encoded antibiotic resistance in various MDR bacterial strains of clinical isolates, suppressing the grow of ***E. coli*** strain is resistant to ampicillin, gentamycin, kanamycin, neomycin, ciprofloxacin, cefoperazone, and ceftazidime (Lathaa et al., 2009). Beg et al. revealed that the alcoholic extract of ***Plumbago zeylanica*** (root) exhibited strong antibacterial activity against ***Salmonella paratyphi*** (***S. paratyphi***)**, *S. aureus, E. coli, Shigella dysenteriae*** and a R-plasmid-harboring standard strain, ***E. coli*** x***+***. In addition, the TCM extracts could induce R-plasmid elimination from ***E. coli*** x ***+***(pUK 651) effectively (Beg and Ahmad, 2000; Tables 2, 3).

### Effect on the Permeability of Cell Membrane

Traditional Chinese medicine can change some ion channels and the permeability of cell membranes to transport antibiotics into bacterial through bacterial cell wall, diminishing antibiotic resistance. Chelerythrine isolated from the root of *Toddalia asiatica (Linn) Lam* disclose strong antibacterial activities against *S. aureus*, *MRSA*, and extend spectrum BLs *S. aureus* (He N. et al., 2018). Scanning electron microscope results revealed the morphological changes in chelerythrine-treated bacteria, such as the damage of both cell wall and cell membrane and the destruction of the channels across the bacterial cell membranes, which allowed protein to leak out of the cell and suppressed protein biosynthesis. Zhao et al. (2019) investigated the antibacterial action of Rhodomyrtosone B from the leaves of *Rhodomyrtus tomentosa* against *MRSA* and vancomycin-resistant *E. faecium*. They found that Rhodomyrtosone B induced the increase on both the perturbation of bacterial membrane potential and membrane permeability to result in its antibacterial. Li et al. also found *Psoralea corylifolia* seed ethanol extract (PCEE) against *MRSA* and *Listeria monocytogenes.* The results from scanning electron microscopy demonstrated that PCEE-treated cells displayed disrupted membranes, incomplete and deformed shapes, indicating that PCEE damaged cell cytoplasmic membranes (Li et al., 2019).

### Inhibition on the Efflux Pump of Antibiotic-Resistant Bacteria

Antibiotics can easily induce the overexpression of bacterial efflux pump to force bacteria pump out more antibacterial drugs to significantly decrease drug concentration at the target site, exacerbating bacterial infection (Paula et al., 2016). The efflux system is observed to be present in both G + and G- bacteria. For example, *MRSA* up-regulates the expression of *NorA* gene to increase drug excretion (Costa et al., 2019). *NorA* efflux pump belongs to MFS family and is first found in clinic to be the important mechanism of bacteria resistant to quinolone and methicillin. Many TCM herbs were efflux pump inhibitors of bacteria that were used to eliminate antibiotic resistance (Tanaka et al., 2016).

Isovalerylshikonin isolated from *Arnebia euchroma (Royle) Johnst (A. euchroma)* was shown to inhibit the bacterial efflux and the expression of MSRA mRNA significantly (He J. M. et al., 2018). Silybin, a flavonolignan component of the extract from the *Silybum marianum (L.) Gaertn.* (*Milk Thistle* seed), prevents the efflux of ciprofloxacin from *MRSA* and blocks the expression of both quinolone-resistant protein *NorA* and quaternary ammonium-resistant proteins *A/B* efflux genes in *MRSA* to restore *MRSA* sensitivity to antibiotics (Wang et al., 2018). Ten batches of *Rhizoma coptidis* display anti-*MRSA* activity on both the *NorA*-negative *S. aureus* strain and the strain that contain a *NorA* gene, using the *MRSA* strain ATCC43300 (contain the *MecA* gene but not the *NorA* gene) and a wild *S. aureus* strain (contain both the *MecA* gene and *NorA* gene). Furthermore, the plectrum-effect relationship analysis reveal that the alkaloid fraction is the main active fraction, and the major constituents with anti-*MRSA* efficacy are berberine, coptisine, palmatine, epiberberine, and jatrorrhizine (Luo et al., 2014). The dichloromethane extract from the Heartwood of *Pilgerodendron uviferum* was shown to block *EtBr* efflux by *S. aureus* strains K2378, which overexpressed the *NorA* gene (*NorA* ++; Espinoza et al., 2019).

The *MexXY-OprM* multidrug efflux system, the first efflux system found in *P. aeruginosa*, is regulated by the *MexZ* repressor and the *oprM* gene, serving as the outer membrane component of several multidrug efflux systems in *P. aeruginosa* (Poole et al., 2015). Su and Wang (2017) demonstrated that Berberine (1/4 MIC) combined with imipenem (1/8 MIC) decreased *MexZ*, *MexX*, *MexY* and outer membrane protein *OprM* to block the *MexXY-OprM* efflux pump in *P. aeruginosa*. Some of TCM herbs are also involved in the inhibition of other efflux pumps. The multidrug efflux pump *EmrD-3* from *Vibrio cholerae* confers the resistance to multiple antimicrobials. *Allium sativum* extract and allyl sulfide also inhibit ethidium bromide efflux in cells, harbor *EmrD-3* and lower the MICs of multiple antibacterial (Bruns et al., 2017). In addition, the multiple roles of cumin against *MRSA* were involved in the disruption of the cell membrane and the inhibition of the *LmrS* efflux pump (Kakarla et al., 2017).

### Modifications of the Antibiotic Molecule

β-lactam antibiotics are bactericidal agents that interrupted bacterial cell wall formation as a result of covalent binding to essential penicillin-binding proteins (PBPs), which are enzymes that are involved in the terminal steps of peptidoglycan cross-linking in both G + and G- bacteria (Bush and Bradford, 2016). Aqueous extracts of *Camellia Sinensis* significantly enhance the activities of imipenem, meropenem, and flucloxacillin against *MRSA* isolates to reduce β-lactam antibiotic resistance in *MRSA* (Stapleton et al., 2004). Yu et al. (2005) revealed that Berberine, the main antibacterial substance of *Coptidis rhizoma* (Coptis chinensis Franch) and *Phellodendri cortex* (Phellodendron amurense Ruprecht) exhibited antimicrobial activity against clinical isolates of *MRSA* through restoring the effectiveness of β-lactam antibiotics against *MRSA*, inhibiting the *MRSA* adhesion and invading intracellularly into human gingival fibroblasts (HGFs).

### Changes in Drug Targets

Peptidoglycan is the major component of the cell envelope of most bacteria. In peptidoglycan synthesis, a number of proteins such as *Mur* enzymes and PBPs were found to be the targets of antibiotics (Liu and Breukink, 2016). Changes in the structure and quantity of PBPs play important roles in bacterial drug resistance. Penicillin binding protein 2′ (2a; PBP2a) in *MRSA* cells containing Corilagin or Tellimagrandin I almost loses the ability to bind benzyl penicillin (Shiota et al., 2004). Corilagin and Tellimagrandin I isolated, respectively, from the extract of *Arctostaphylos uvaursi* and *Rosa canina L*, diminish the binding activity of PBP2 and PBP3, resulting in a remarkable reduction in the resistance level of β-lactams in *MRSA*. Meng et al. demonstrated that 139 constituents of the *Verbena officinalis*, *Magnolia officinalis*, *Momordica charantia*, and *Daphne genkwa* could bind to two *MRSA* blockage targets (penicillin binding proteins PBP2a and PBP4) to reduce the resistance. Among those constituents, *Pinoresinol*, *Tiliroside*, *Momorcharaside B*, and *Magnatriol B* had been confirmed to be the inhibitors of PBP2a or PBP4 (Kuok et al., 2017).

### Effects on the BBF

Bacterial biofilm formation and QS may serve as a promising therapeutic alternative to combat MDR pathogens. TCM herbs and their active components can block the BBF formation and QS system to improve the bacteriostatic and bactericidal effects of antibiotics.

#### Inhibition on the BBF Formation

Bacteria formed BBFs, in which the bacteria are wrapped to form the membrane to increases the resistance of antimicrobial agents (Singh et al., 2017). The biofilm formation is present in a variety of bacteria, including *P. aeruginosa*, *S. epidermidis*, and *E. coli* (May et al., 2009; Sharma et al., 2016).

Bacterial biofilm formation involves protein complex which include amyloid fibers, exopolysaccharides and extracellular DNA in a self-produced extracellular polymeric matrix (Singh et al., 2017). Serra et al. (2016) revealed that epigallocatechin gallate extracted from green tea abolished amyloid curly fiber production to suppress BBF formation through utilizing bacterial macro-colony biofilms of commensal and pathogenic *E. coli* as one model system. *Herba patriniae* (*H. patriniae*) extract is able to reduce the biofilm formation significantly and alter the structure of the mature biofilms of *P. aeruginosa* dramatically, further decreasing exopolysaccharide production of *P. aeruginosa* and promoting its swarming motility (Fu et al., 2017). Ginger extracts were also shown to inhibit the biofilm formation of *P. aeruginosa* through another mechanism decreasing the production of extracellular polymeric substances (Kim and Park, 2013).

There are several stages in biofilm formation, including reversible adhesion, irreversible adhesion, colony-forming stage and biofilm mature stage. The ethanol extract of *Sanguisorba officinalis L.* is able to increase the transcript level of *icaR* to decrease the transcript levels of the *icaADBC* operon which encodes the polysaccharide intercellular adhesion synthetases, further inhibiting the biofilm formation of *MRSA*. These results indicated that *Sanguisorba officinalis L.* inhibited biofilm formation of *MRSA* in an *ica*-dependent manner (Chen et al., 2015). In addition, *Berberis aristata* is also confirmed to exhibit the maximum potential in some activities against biofilm formation, such as anti-adhesion and anti-QS, respectively. *Camellia sinensis* displays both anti-adhesion and anti-QS potential while *Holarrhena antidysenterica* only exhibits anti-QS potential. Therefore, both *Berberis aristata* and *Camellia sinensis* are potent herbs with significant therapeutic potential (Thakur et al., 2016; Table 3).

#### Anti-QS Activity

Quorum Sensing regulates various bacterial behaviors, such as the formation of biofilm and the secretion of virulence factors (such as elastase, rhamnolipid, and pyocyanin; Lou et al., 2017). QS inhibitors are able to interfere with the QS system of pathogens and reduce the pathogenic effects, However, the majority of QS inhibitors have been found to be toxic, and cannot be used in medicine fields. Screening QS inhibitors from TCM resources has become a new strategy for the development of natural antibacterial agents (Khider et al., 2019). Both *Berberine* and *Matrine* are able to inhibit biofilm formation of antimicrobial-resistant *E. coli* strains through downregulating the QS system, and *Berberine* is more effective than Matrine (Sun et al., 2019).

*Lagerstroemia speciosa (Lythraceae)* fruit extracts downregulate QS-related genes (*Las* and *Rhl)* and signaling molecules (such as *N-acylhomoserine lactones*) to enhance bacterial susceptibility to Tobramycin (Singh et al., 2012). The methanol extracts of *Rhizophora apiculata* and *Rhizophora mucronata* (1 mg/ml) exhibit significant inhibition on QS dependent virulence factors production, such as *Las A protease*, *LasB elastase*, *total protease*, pyocyanin pigment production and biofilm formation in P. aeruginosa PAO1 and clinical isolates (*CI-I* and *CI-II*; Annapoorani et al., 2013). In addition, Koh and Tham (2011) screened many TCM herbs and found eight of them as QS inhibitors: *Prunus armeniaca, Prunella vulgaris, Nelumbo nucifera, Panax notoginseng* (root and flower), *Punica granatum, Areca catechu, and Imperata cylindrica* (Tables 2, 3).

## Evaluation of TCM Against Antibiotic-Resistant Bacteria in Mouse Models

The feasibility of TCM against antibiotic-resistant bacteria has been shown *in vitro* experiments. With the further investigations on antibacterial mechanism of TCM, the analysis of antibacterial components of TCM and verification of the blockage effect of TCM *in vivo* contribute to develop TCM resources with antibacterial activities. Preclinical investigations in mouse models is an essential step for clinical application of TCM herbs and their ingredients, and those experimental results have shown the bacteriostatic and actual effect of those TCM herbs *in vivo* (Table 4).

**TABLE 4:** Evaluation of TCM active ingredients against antibiotics-resistant bacteria in mouse models.

**Mouse models**	**Active against**	**Compounds**	**Mechanism of action**	**References**
Wound model	*P. aeruginosa*	Chlorogenic acid	To inhibit the formation of biofilm	Wang et al., 2019
Pneumonia model	*A. baumannii*	Extracts of *Scutellaria* barbata	To be studied	Tsai et al., 2018
Peritonitis/sepsis model	*S. aureus*	Isova shikonin	To prevent the formation of biofilm	Yang et al., 2018
Lethal pneumonia model	*MRSA*	Baicalein	To block the coagulase activity of von Willebrand factor-binding protein	Zhang et al., 2020
Skin infection model	*MRSA and VRE*	Rhodomyrtosone B from *Rhodomyrtus tomentosa*	To increase membrane permeability	Zhao et al., 2019
Pneumonia model	*MRSA*	Ethyl acetate fraction (S20b) from *Pithecellobium clypearia*	To damage the cell wall and increase cell membrane permeability.	Liu et al., 2020

Tsai et al. screened 30 TCM herbs prescribed for heat-clearing and detoxifying to analyze their antibacterial activities in lung infection mouse models through disk diffusion assays and time-kill assays, indicating that *Scutellaria barbata* could be used as an alternate drug to treat *A. baumannii*-resistant pulmonary infections (Tsai et al., 2018). Yang et al. revealed that Isovalerylshikonin isolated from TCM herb *Arnebia Euchroma*, displayed marginal antibacterial activity against antibiotic-resistant *S. aureus*. The synergistic effects between Isovalerylshikonin and streptomycin were obtained by using sepsis mouse model (Yang et al., 2018). Ethyl acetate fraction from *Pithecellobium clypearia* was found to exhibit therapeutic potential for *MRSA* pneumonia in pneumonia mouse model. S20b damaged the *MRSA* cell wall and promoted more potassium ions to flow out of the cells to increase the permeability of cell membrane *in vivo* (Liu et al., 2020). In addition, *Baicalein* isolated from *Scutellaria baicalensis*, is found to attenuate the virulence of *S. aureus* and protects mice from *S. aureus* induced lethal pneumonia through blocking the coagulase activity of von Willebrand factor-binding protein in *S. aureus* (Zhang et al., 2020). In wound mouse model, *Chlorogenic acid* (an active component of some TCM herbs) treatment is able to accelerate mouse healing rate and decrease the bacteria number in wound areas (Wang et al., 2019). Therefore, the suppressive mechanism of TCM herbs and their ingredients on antibiotic-resistant bacteria have been investigated broadly in order to develop clinical application of TCM bacteriostatic products.

## Future Prospect

There has been an emerging trend of multi-target drug development, using TCM herbs and their active ingredients. With the in-depth research of the mechanism of multi-drug resistance, studies related to the antibacterial mechanism of TCM, such as effective bacteriostatic ingredients of TCM, continuous progress of separation and purification techniques, and the application of TCM in drug-resistant bacteria inhibition, have been performed progressively. The extraction and isolation of antibacterial active ingredients or compounds of TCM and its active ingredients in combination with antibiotics enhance the antibacterial effect of antibiotics, which has become a novel antibacterial treatment measure and has been widely recognized by healthcare experts.

## Author Contributions

ZQ and PQ conceived and designed the work. ZQ, HL, and DL coordinated technical support and funding. TS and PQ wrote the manuscript. BY, YQ, and XH performed tables and figure. All authors contributed to the article and approved the submitted version.

## Conflict of Interest

The authors declare that the research was conducted in the absence of any commercial or financial relationships that could be construed as a potential conflict of interest.

## Abbreviations

*A. baumannii*: *Acinetobacter baumannii*
*C. albicans*: *Candida albicans*
*C. violaceum*: *Chromobacterium violaceum*
*C. gloeosporioides*: *Colletotrichum gloeosporioides*
*E. faecalis*: *Enterococcus faecalis*
*E. coli*: *Escherichia coli*
*L. monocytogenes*: *Listeria monocytogenes*
*MRSA*: *Methicillin-resistant Staphylococcus aureus*
*M. tuberculosis*: *Mycobacterium tuberculosis*
*P. shigelloides*: *Plesiomonas shigelloides*
*P. aeruginosa*: *Pseudomonas aeruginosa*
*R-*: *resistant strain*
*S. typhi*: *Salmonella typhi*
*S. boydii*: *Shigella boydii*
*S. dysenteriae*: *Shigella dysenteriae*
*S. aureus*: *Staphylococcus aureus*
*S. epidermidis*: *Staphylococcus epidermidis*
*S. faecalis*: *Streptococcus faecalis*
*S. mitis*: *Streptococcus mitis*
*S. mutans*: *Streptococcus mutans*
*S. pneumoniae*: *Streptococcus pneumoniae*
*V. cholerae*: *Vibrio cholerae*
*VRE*: *vancomycin resistant Enterococcus faecium.*
